# Roles of lipoxin A4 receptor activation and anti-interleukin-1β antibody on the toll-like receptor 2/mycloid differentiation factor 88/nuclear factor-κB pathway in airway inflammation induced by ovalbumin

**DOI:** 10.3892/mmr.2015.3443

**Published:** 2015-03-05

**Authors:** XIA KONG, SHENG-HUA WU, LI ZHANG, XIAO-QING CHEN

**Affiliations:** 1 Department of Pediatrics, The First Affiliated Hospital of Nanjing Medical University, Nanjing, Jiangsu 210029, P.R. China; 2 Department of Pediatrics, Nanjing First Hospital, Nanjing Medical University, Nanjing, Jiangsu 210006, P.R. China

**Keywords:** lipoxin A4, ovalbumin, toll-like receptor 2, mycloid differentiation factor 88, nuclear factor-κB, interleukin-1β

## Abstract

Previous studies investigating the role of toll-like receptors (TLRs) in asthma have been inconclusive. It has remained elusive whether the toll-like receptors (TLR2)/mycloid differentiation factor 88 (MyD88)/nuclear factor (NF)-κB signaling pathway is involved in lipoxin A4 (LXA4)-induced protection against asthma. Therefore, the present study investigated whether ovalbumin (OVA)-induced airway inflammation is mediated by upregulation of the TLR2/MyD88/NF-κB signaling pathway, and whether it proceeds via the inhibition of the activation of the LXA4 receptor and anti-interleukin (IL)-1β antibodies. Mice with airway inflammation induced by OVA administration were treated with or without a LXA4 receptor agonist, BML-111 and anti-IL-1β antibody. Serum levels of IL-1β, IL-4, IL-8 and interferon-γ (IFN-γ) were assessed, and levels of IL-1β, IL-4, IL-8 and OVA-immunoglobulin (Ig)E, as well as leukocyte counts in the bronchoalveolar lavage fluid (BALF) were measured. Pathological features and expression of TLR2, MyD88 and NF-κB in the lungs were analyzed. Expression of TLR2 and MyD88, and activation of NF-κB in leukocytes as well as levels of IL-4, IL-6 and IL-8 released from leukocytes exposed to IL-1β were assessed. OVA treatment increased the levels of IL-1β, IL-4 and IL-8 in the serum and BLAF, the number of leukocytes and the levels of OVA-IgE in the BALF, the expression of TLR2 and MyD88, and the activation of NF-κB in the lung. These increments induced by OVA were inhibited by treatment with BML-111 and anti-IL-1β antibodies. Treatment of the leukocytes with BML-111 or TLR2 antibody, or MyD88 or NF-κB inhibitor, all blocked the IL-1β-triggered production of IL-4, IL-6 and IL-8 and activation of NF-κB. Treatment of the leukocytes with BML-111 or TLR2 antibody suppressed IL-1β-induced TLR2 and MyD88 expression. The present study therefore suggested that OVA-induced airway inflammation is mediated by the TLR2/MyD88/NF-κB pathway. IL-1β has a pivotal role in the airway inflammation and upregulation of the TLR2/MyD88/NF-κB pathway induced by OVA. BML-111 and anti-IL-1β antibody restrains the OVA-induced airway inflammation via downregulation of the TLR2/MyD88/NF-κB pathway.

## Introduction

Asthma is a chronic inflammatory disorder of the airways, and is characterized by reversible bronchoconstriction, airway hyper-responsiveness and airway remodeling (1). Various cells that migrate from the bloodstream to the bronchial tree are sources of local inflammation. Numerous eosinophils, T lymphocytes and polymorphonuclear cells (PMNs) infiltrate peribronchial tissues in asthmatics, introducing into the lungs an increased capacity to generate proinflammatory mediators, cytokines and chemokines, including leukotriene (LT) B4, cysteinyl leukotrienes (CysLTs), T-helper lymphocyte 2 (Th2) cytokines and interleukins (ILs) (2,3). IL-1β has a pivotal role in the progression of allergic airway inflammation in asthma (3,4). Leukotrienes (LTs) are lipids synthesized from arachidonic acids by mast cells, eosinophils and alveolar macrophages in the lungs, and have been considered to have an important role in the pathogenesis of asthma (2). LTB4 acts as a chemoattractant pro-adhesive agent and secretagogue for PMNs, eosinophils and T lymphocytes (4,5). CysLTs cause bronchoconstriction, which contributes to the responses to an inhaled allergen challenge (6). In contrast to proinflammatory LTs, lipoxins, a separate class of eicosanoids that are distinct in structure and function from CysLTs and LTB4, act as potent anti-inflammatory agents (7). Lipoxin A4 (LXA4) and its analogs inhibit LTB4-induced PMN and eosinophil chemotaxis (8). A previous study by our group also demonstrated that LXA4 suppressed the production of LTB4 in PMNs (9). In addition, LXA4 and its analogs blocked CysLTs-triggered airway obstruction and hypersecretion of CysLTs in bronchoalveolar lavage fluids (BALF) obtained from asthmatic mice (10,11). Furthermore, LXA4 analogs inhibited airway hyper-responsiveness and pulmonary inflammation in murine models of asthma, as shown by decreased leukocytes and mediators in BALF, including IL-5, IL-13, eotaxin, prostanoids and CysLTs (11). Moreover, transgenic overexpression of LXA4 receptor in leukocytes obtained from asthmatic mice led to a significant inhibition of pulmonary inflammation and eosinophil infiltration in the lungs (11). In addition, LXA4 repressed the levels of IL-8 released from peripheral blood mononuclear cells obtained from patients with asthma (12). To date, the signaling pathways involved in LXA4 action in asthmatic models remain to be fully elucidated. A previous study by our group showed that LXA4 abrogated the synthesis of IL-1β, IL-6 and IL-8 induced by lipopolysaccharide (LPS) via downregulation of nuclear factor (NF)-κB pathway-dependent signal transduction in pulmonary microvascular endothelial cells (13). LPS activates NF-κB through toll-like receptor (TLR) 4 and mycloid differentiation factor 88 (MyD88) in endothelial cells (14). In this regard, increasing evidence suggested that toll-like receptors (TLRs) may be potential mediators or modulators of inflammation within the lungs (15–17). TLRs are the best characterized class of pattern recognition receptors of the innate immune system, and trigger anti-microbial host defense responses (18). Various cells, including leukocytes and airway epithelial cells, express TLRs (16). Signaling through the TLRs leads to transcription and translation of a variety of cytokines and mediators (16). However, previous studies investigating the role of TLRs in asthma have been inconclusive. For example, Redecke *et al* (17) demonstrated that activation of TLR2 induced a Th2 immune response and promoted experimental asthma. Conversely, Velasco *et al* (19) reported that TLR4 and TLR2 agonists decreased allergic inflammation. Therefore, the present study was designed to examine the changes in the TLR2/MyD88/NF-κB signaling pathway in asthmatic mice, and also to investigated whether the TLR2/MyD88/NF-κB signaling pathway is involved in the inhibitory effects of LXA4 on pulmonary inflammation in asthmatic mice, and to determine whether IL-1β modulates the changes in the TLR2/MyD88/NF-κB signaling pathway in asthmatic mice.

LXA4 action is mediated by the LXA4 receptor (ALX) expressed on the membrane of various cell types, including airway epithelial cells and leukocytes, and ALX can be upregulated by specific inflammatory mediators (7). Allergen sensitization and challenge with ovalbumin (OVA) increases ALX expression in infiltrating leukocytes and airway epithelial cells in the lungs of asthmatic mice (11). Following stimulation by mediators, LXA4 is rapidly generated at sites of inflammation, acts locally and is then rapidly inactivated by metabolic enzymes (7). Thus, the use of LXA4 may not be suitable for *in vivo* experiments. Instead, stable analogs of LXA4 and LXA4 receptor agonist, including BML-111 and CGEN-855A, were used for *in vivo* experiments (10,11,20–22). Accordingly, the present study used BML-111, a potent ALX agonist with an inhibitory activity on LTB4-induced PMN chemotaxis similar to that of LXA4 (21), was used in the *in vivo* experiment.

## Materials and methods

### Animals

Male BALB/c mice weighing 19–21 g were obtained from the Laboratory Animal Center of Nanjing First Hospital (Nanjing, China), and quarantined for one week prior to the experiment and bled to establish that they were virus free. The mice were housed in the animal facility that was maintained at 22–24°C with a 12-h dark/light cycle, and fed with commercial pelleted mouse food and water *ad libitum* under specific pathogen-free conditions. The present study was performed in strict accordance with the recommendations in the Guide for the Care and Use of Laboratory Animals of the National Institutes of Health. The protocol was approved by the Committee on the Ethics of Animal Experiments of Nanjing First Hospital affiliated to Nanjing Medical University (permit number, 2013-6135). All surgical procedures were performed under sodium pentobarbital (Sigma-Aldrich, St. Louis, MO, USA) anesthesia, and all efforts were made to minimize suffering.

### Induction of asthmatic models

The mice were randomly divided into six groups, i.e., normal controls (NC), asthmatic mice (AM), BML-111-treated asthmatic mice (BAM), vehicle (0.1 ml of ethanol) of BML-111-treated asthmatic mice (VAM), anti-IL-1β antibody-treated asthmatic mice (AAM) and rabbit immunoglobulin (Ig)G-treated asthmatic mice (RAM). Each group consisted of 10 mice, and 5 mice were used for BALF collection, another 5 mice were used for blood collection and pathologic studies. For induction of asthmatic models, BALB/c mice were sensitized with 10 *μ*g of OVA (Sigma-Aldrich) emulsified in 20 mg aluminum hydroxide (Sigma-Aldrich) as adjuvant in a total volume of 0.1 ml of normal saline by intraperitoneal injection at days 0, 7 and 14. Subsequently, the OVA challenges were given once a day at days 25, 27, 29, 31 and 33 with 1% aerosolized OVA in saline for 30 min using an atomizer (PARI BOY N085; PARI GmbH, Starnberg, Germany). The mice were sacrificed 24 h after the final OVA challenge by cervical dislocation. The NC mice were sensitized and challenged by using the same protocol but using saline instead of OVA. The mice from the AM, VAM and RAM groups but not those from the NC, BAM or AAM groups, had the asthmatic symptoms, including dysphoria, short breath, tachypnea and apathism from days 31–33.

### BML-111 treatment of asthmatic mice

BML-111 (5*S*,6*R*, 7-trihydroxyheptanoic acid methyl ester; Cayman Chemicals, Ann Arbor, MI, USA) was diluted in ethanol (Sigma-Aldrich) at a concentration of 200 *μ*g/ml. Following the final OVA sensitization, the BAM mouse was intraperitoneally injected with 0.1 ml BML-111 at a dose of 1 *μ*g/g body weight (or 0.1 ml ethanol for the VAM mouse) 30 min prior to the OVA challenges at days 25, 27 and 29. Twenty-four hours following the final OVA challenge, the mouse was anesthetized by an intraperitoneal injection of 0.6 mg of sodium pentobarbital. Subsequently, the blood and BALF were collected and stored at −20°C until use. The lungs were removed and excised. The low lobes of the left lungs were processed for western blot analysis and electrophoretic mobility shift assay (EMSA), while the low lobes of the right lungs were fixed with 4% paraformaldehyde for 24 h and then stained for pathological examination.

### Anti-IL-1β antibody treatments of asthmatic mice

Rabbit anti-mouse IL-1β antibody (Thermo Fisher Scientific, Waltham, MA, USA) was diluted in sterile water. After the final OVA sensitization, mice in the AAM group were subcutaneously injected with 1 *μ*g/g body weight of anti-IL-1β antibody in a total volume of 0.1 ml sterile water 30 min prior to the OVA challenges at days 25, 27, 29, 31 and 33. Twenty-four hours after the final OVA challenge, the mice were sacrificed and the samples were collected in the same way as mentioned above. Mice in the RAM group were treated by using the same protocol but using rabbit immunoglobulin (Ig)G (1 *μ*g/g, Santa Cruz Bio Biotechnology, Inc., Dallas, TX, USA) instead of anti-IL-1β antibody.

### ELISA of IL-1β, IL-4, IL-8 and interferon-γ (IFN-γ)

Serum levels of IL-1β, IL-4, IL-8 and IFN-γ were measured using ELISA kits (USCN Life Science, Wuhan, China) following the manufacturer’s instructions. The limits of detection for IL-1β, IL-4, IL-8 and IFN-γ by the ELISA were 1 pg/ml.

### Leukocyte counts and ELISA of BALF

Bilateral BALF was obtained by four injections of 1 ml saline through a tracheal cannula into the lungs and four repeats of bronchial lavage. The BALF, obtained from each mouse at an average of 3.5 ml, was centrifuged and resuspended as two aliquots of 1 ml of phosphate-buffered saline (PBS; Sigma-Aldrich) plus 0.6 mM EDTA (Sigma-Aldrich). Subsequently, the cells in BALF were enumerated and identified following Wright-Giemsa (Sigma-Aldrich) staining with a hemocytometer, and the leukocytes were differentiated into eosinophils, neutrophils and lymphocytes according to standard cellular morphology and staining characteristics. The leukocytes were counted by two independent investigators in a single-blinded study, which analyzed at least 200 leukocytes for each mouse from four different random locations using a microscope (CX22; Olympus Corporation, Tokyo, Japan). The levels of IL-1β, IL-4, IL-8 and OVA-specific IgE in BALF were determined using ELISA kits (for ILs, USCN Life Science, Wuhan, China; for OVA-IgE, Shibayagi, Gunma, Japan) according to the manufacturer’s instructions.

### Lung tissue pathological studies

The lung sections were stained with hematoxylin and eosin (Sigma-Aldrich) and observed using light microscopy. Assessment of grades of bronchial and peribronchial inflammation was based on a six-scale score as previously described (23). Expressions of TLR2 and phosphorylated NF-κB p65 were determined using immunohistochemical staining. Briefly, the sections of lungs were deparaffinized and treated with hydrogen peroxide to block endogenous peroxidase activity. Sections were incubated with either rabbit anti-mouse polyclonal TLR2 antibody (cat. no. sc-10739) or rabbit anti-mouse polyclonal phosphorylated NF-κB p65 (Ser 276) antibody (cat. no. sc-109; Santa Cruz Biotechnology, Inc.) at 1:100 dilution. Subsequently, biotinylated goat anti-rabbit IgG antibody (Vector Labs, Burlingame, CA, USA) was applied. The sections were exposed to avidin-biotinylated horseradish peroxidase (HRP) and diaminobenzidine tetrahydrochloride (Vector Labs). Hematoxylin staining was used for counterstaining. The mean ratios of TLR2- and NF-κB-positive cellular areas (deep brown) in five fields of view per section from each mouse were assessed by a JD-801 computer-aided image analyzer (Jeda Co., Nanjing, China) under high power magnification (x200; Olympus CX22).

### Western blot analysis of TLR2 and MyD88

The lung tissues were homogenized in lysis buffer with protease inhibitors. Proteins in the lysates were extracted using Protein Extraction kits (Active Motif, Carlsbad, CA, USA) according to the manufacturer’s instructions. After total protein was measured using the Bradford method, 10 *μ*l lysate was purified using 10% SDS-PAGE for 4 h prior to blotting onto polyvinylidene difluoride membranes (Amersham, Arlington, IL, USA). Non-specific sites on the membranes were blocked with 5% non-fat milk (Sigma-Aldrich). The blots were incubated with primary rabbit anti-mouse polyclonal antibodies against TLR2 (cat. no. sc-10739), MyD88 (cat. no. sc-11356) or β-actin (cat. no. sc-130656; Santa Cruz Biotechnology, Inc.) at 1:2,000 dilution overnight followed by incubation for 2 h with an HRP-conjugated goat anti-rabbit IgG antibody (Jackson, West Grove, PA, USA) at 1:5,000 dilution. Following washing, the membranes were incubated with an enhanced chemiluminance reagent system (Amersham) and then exposed to Kodak Biomax films (Eastman Kodak, Rochester, NY, USA). Semiquantitative analysis was performed using UVP-gel densitometry (UVP Co., San Gabriel, CA, USA). Arbitrary unit = (A_TLR2_/A_β-actin_) ×100%, or (A_MyD88_/A_β-actin_) ×100%.

### EMSA of NF-κB activation

Nuclear proteins were extracted using a Nuclear Protein Extraction kit (Active Motif). EMSA was performed using a Gel Shift Assay kit (Promega, Madison, WI, USA) following the manufacturer’s instructions. The nuclear extracts containing 30 *μ*g of total protein were pre-incubated with gel shift binding buffer for 10 min, followed by the addition of γ-(^32^P)-labeled double-stranded oligonucleotide probes of NF-κB (Santa Cruz Biotechnology, Inc.) and further incubated for 20 min in a binding reaction mixture. The oligonucleotide pairs for NF-κB were 5′-AGTTGAGGGGACTTTCCCAGGC-3′ and 5′-GCCTGGGAAAGTCCCCTCAACT-3′. Formed nuclear protein-DNA complexes were dissolved in 4% non-denaturing polyacrylamide gels. Electrophoresis was performed under 90 V for 2 h. The gels were dried and exposed to Kodak X-ray films (Eastman Kodak) at −70°C for 36 h. Semiquantitative analysis was performed using UVP-gel densitometry. Arbitrary unit = (A_NC_ or A_AM_ or A_BAM_ or A_VAM_ or A_AAM/Aunlabeled probes_) ×100%. To assess the specificity of the reaction, competition assays were performed with 100-fold excess of unlabeled consensus oligonucleotide pairs of NF-κB, which were added to the binding reaction mixture 10 min prior to the addition of the labeled probes.

### ELISA of IL-4, IL-6 and IL-8 released from leukocytes

A whole blood sample was obtained from an NC mouse and drawn into 5-ml tubes containing heparin (Sigma-Aldrich). Leukocytes were separated from the blood as previously described (9). Following washing with PBS, the leukocytes were resuspended and adjusted to a density of 2×10^6^ cells/ml in RPMI-1640 supplemented with 0.5% FCS and antibiotic-anti-mycotic solution (Gibco-BRL, Invitrogen Life Technologies, Inc., Carlsbad, CA, USA). The cells were then seeded into a 24-well plate in 1 ml medium and cultured at 37°C in a 5% CO_2_ incubator. Subsequently, the cells were stimulated with IL-1β (10 ng/ml; Sigma-Aldrich) with or without pretreatment with BML-111 (1 mM) for 30 min, *N*-t-Boc-Phe-Leu-Phe-Leu-Phe (BOC1, an antagonist of G protein-coupled LXA4 receptor; 100 *μ*M; Calbiochem, San Diego, CA, USA) for 30 min, goat anti-mouse TLR2-neutralizing polyclonal antibody (TLR2Ab; 1 *μ*g/ml; Santa Cruz Biotechnology, Inc.) for 1 h, goat IgG (1 *μ*g/ml; Santa Cruz Biotechnology, Inc.) for 1 h, ST2825 (a MyD88 dimerization inhibitor; 20 *μ*M; MedChem Express, Princeton, NJ, USA) for 1 h, and thiophene-3-car-boxamide 1 (TPCA-1; an inhibitor of IκB kinase β; 10 *μ*M; Sigma-Aldrich) for 1 h. Following co-incubation for 24 h, the cellular supernatants were collected for measurement of IL-4, IL-6 and IL-8 proteins using ELISA kits (USCN Life Science) following the manufacturer’s instructions. The cell viability was measured by trypan blue exclusion assay (Sigma-Aldrich) in pilot experiments and the percentage of viable cells was >95% following exposure to the abovementioned mediators.

### Western blot analysis of TLR2 and MyD88 in leukocytes

The leukocytes were treated with the mediators in the same way as mentioned above. Following co-incubation for 30 min, the cells were collected, total protein in the lysates was extracted, and the expression of TLR2, MyD88 and β-actin were assessed with using western blot analysis in a similar way to the abovementioned procedure.

### Determination of NF-κB activation in leukocytes

The leukocytes were treated with the mediators in the same way as mentioned above. Following co-incubation for 1 h, the cytosolic or nuclear extracts were obtained using NE-PER Nuclear and Cytoplasmic Extraction reagents (Fisher Thermo Scientific) in the presence of protease inhibitors and phosphatase inhibitor (Roche Diagnostics, Indianapolis, IN, USA). Denatured proteins were purified using 10% SDS-PAGE (Amersham). Following electrophoresis, separated proteins were transferred to polyvinylidene difluoride membranes (Amersham). Nonspecific sites were blocked with 5% nonfat milk for 2 h. Subsequently, the cytosolic protein blots were incubated with rabbit anti-mouse NF-κB p65 or IκBα or anti-GAPDH antibodies (Cell Signaling Technology, Beverly, MA, USA), and the nuclear protein blots were incubated with rabbit anti-mouse phosphorylated NF-κB p65 (Ser 276) antibody (Santa Cruz Biotechnology, Inc.) or rabbit anti-mouse histone 3 antibody (Cell Signaling Technology) overnight at 4°C. The blots were then incubated for 2 h with an HRP-conjugated goat anti-rabbit IgG antibody (Jackson, West Grove, PA, USA) at 1:5,000 dilution. Following washing, the membranes were incubated with an enhanced chemilu-minance reagent system (Amersham) and then exposed to Kodak Biomax films (Eastman Kodak). Semiquantitative analysis was performed by using UVP-gel densitometry (UVP, Co.). Arbitrary unit = (A_IκBα_/A_GAPDH_) ×100%, or (A_cytosolic p65_/A_GAPDH_) ×100%, or (A_nuclear p65_/A_histone 3_) ×100%.

### Statistical Analysis

Values are expressed as the mean ± standard deviation (SD). Experimental values are analyzed using one-way analysis of variance followed by F and q tests. The degree of peribronchial inflammation was analyzed using the χ^2^ test. All analyses were performed using the Statistical Package for Social Sciences version 16.0 (SPSS, Inc., Chicago, IL, USA). Differences between values were considered to be statistically significant when P<0.05.

## Results

### Serum IL-1β, IL-4, IL-8 and IFN-γ levels

**Figure 1:**
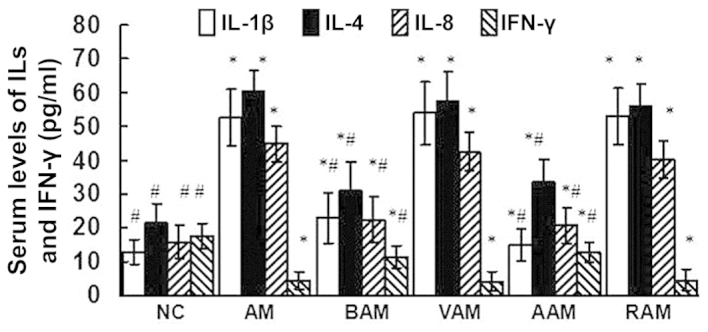
Serum levels of IL-1β, IL-4, IL-8 and IFN-γ assessed using ELISA. Values are expressed as mean ± standard deviation of five mice in each group. ^*^P<0.05, as compared with the levels of the same cytokine in serum obtained from NC mice. ^#^P<0.05, as compared with the levels of the same cytokine in serum obtained from AM mice. NC, normal control; AM, ovalbumin immunization-induced asthmatic mice; BAM, BML-111-treated AM; VAM, vehicle of BML-111-treated AM; AAM, anti-IL-1β antibody-treated AM; RAM, rabbit immunoglobulin G-treated AM; IL, interleukin; IFN, interferon.

As shown in Fig. 1, OVA immunization upregulated the serum levels of IL-1β, IL-4 and IL-8, downregulated the serum levels of IFN-γ, and therefore increased serum IL-4/IFN-γ ratios in mice. However, treatment of asthmatic mice with BML-111 partially but significantly abrogated the abovementioned changes induced by OVA. Treatment with anti-IL-1β antibody significantly reduced the serum levels of IL-1β, IL-4 and IL-8, and augmented the serum levels of IFN-γ reduced by OVA immunization.

### Leukocyte counts, ILs and OVA-IgE in BALF

**Figure 2:**
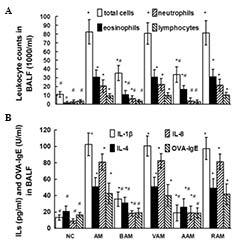
(A) Leukocyte counts and (B) levels of IL-1β, IL-4, IL-8 and OVA-IgE assessed using ELISA in BALF. Values are expressed as the mean ± standard deviation of five mice in each group. ^*^P<0.05, as compared with the number of the same cells in A or levels of the same cytokine in BALF obtained from NC mice in B. ^#^P<0.05, as compared with the number of the same differential cells in A or the levels of the same cytokine in BALF obtained from AM mice in B. NC, normal control; AM, ovalbumin immunization-induced asthmatic mice; BAM, BML-111-treated AM; VAM, vehicle of BML-111-treated AM; AAM, anti-IL-1β antibody-treated AM; RAM, rabbit IgG-treated AM; BALF, bronchoalveolar lavage fluid; Ig, immunoglobulin.

As indicated in Fig. 2A, the number of total leukocytes, eosinophils, neutrophils and lymphocytes increased in the BALF obtained from the AM mice compared to those in normal controls. However, OVA immunization did not affect the number of monocytes/macrophages compared with those in normal controls (data not shown). Treatment with BML-111 partially but significantly suppressed the increments in the number of total leukocytes, eosinophils, neutrophils and lymphocytes in BALF induced by OVA immunization. Similarly, treatment with anti-IL-1β antibody also reduced the number of total leukocytes, eosinophils, neutrophils and lymphocytes in BALF stimulated by OVA immunization. In addition, BML-111 treatment decreased the levels of IL-1β, IL-4, IL-8 and OVA-IgE in BALF induced by OVA (Fig. 2B). Interestingly, treatment with anti-IL-1β antibody also restrained the levels of IL-1β, IL-4, IL-8 and OVA-IgE in BALF induced by OVA (Fig. 2B).

### Lung tissue pathological studies

**Figure 3:**
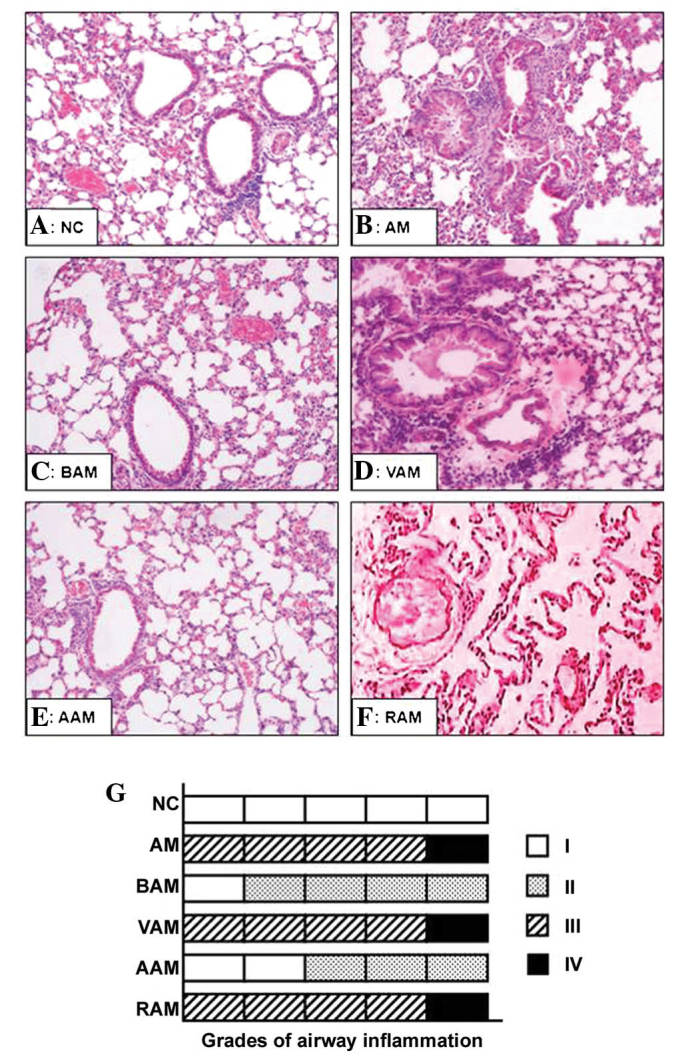
(A–F) Pathological features of lung tissue stained with hematoxylin and eosin from the mice in each group (n=5). (G) Grades of bronchial and perbronchial inflammation were assessed in five mice in each group by using a six-scale score comprising grades I–VI. Grades V and VI were found in all lung tissues. NC, normal control; AM, ovalbumin immunization-induced asthmatic mice; BAM, BML-111-treated AM; VAM, vehicle of BML-111-treated AM; AAM, anti-IL-1β antibody-treated AM; RAM, rabbit immunoglobulin G-treated AM.

As shown in Fig. 3, the lung sections obtained from mice in the AM, VAM and RAM groups exhibited an obvious peribronchial and perivascular inflammatory cell infiltration, as well as a marked cellular swelling, hydropic degeneration, hyperplasia and necrosis in the airway epithelial cells, and an excessive mucus in the lumen of bronchi with thickened bronchial mucosa and smooth muscle as compared with that in normal controls (Fig. 3B, D and F). Treatment with BML-111- and IL-1β antibodies abolished the inflammatory cell infiltration, airway epithelial cell swelling and hydropic degeneration, as well as bronchial mucus hypersecretion induced by OVA (Fig. 3C and E). OVA immunization induced significant airway inflammation, as shown by higher grades of the bronchial and peribronchial inflammation scale (Fig. 3G). However, treatment with BML-111 and anti-IL-1β antibodies significantly inhibited OVA-induced airway inflammation (Fig. 3G; χ^2^=28.14; P<0.001).

### Expression of TLR2 and MyD88

**Figure 4:**
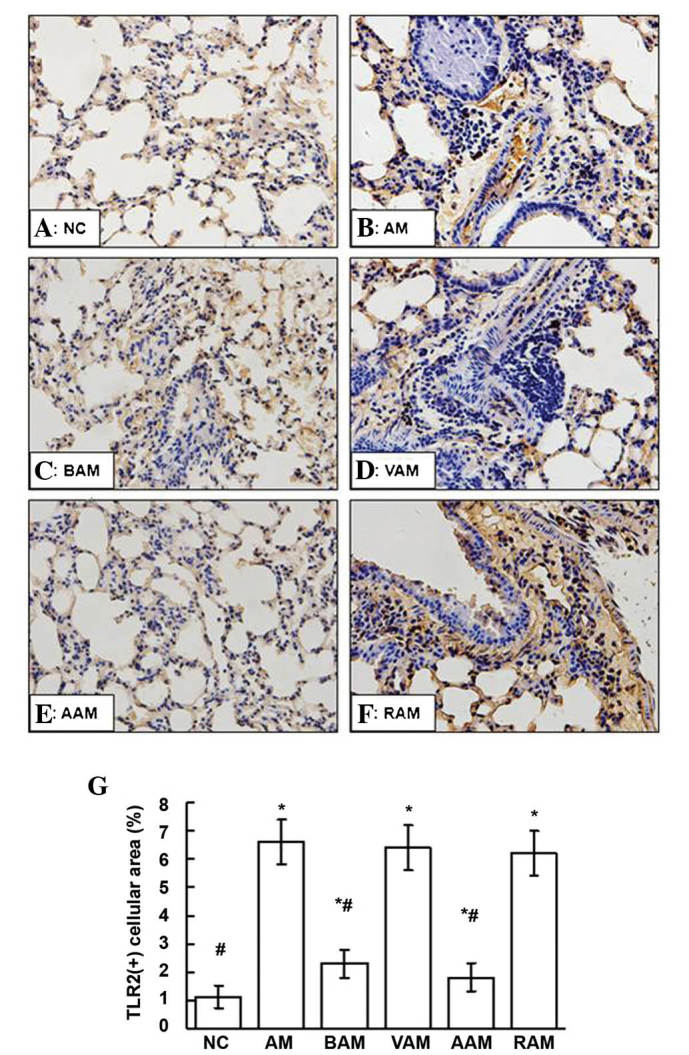
(A–F) Expression of TLR2 in lung tissue from the mice in each group was assessed using immunohistochemistry (deep brown cellular area indicates positive staining; magnification, x200). (G) Quantification of TLR2-positive staining in five fields of view per section from each mouse was assessed by JD-801 computer-aided image analyzer under high-power magnification (x200). Values are expressed as the mean ± standard deviation of five mice in each group. ^*^P<0.05, as compared with the NC group. ^#^P<0.05, as compared with the AM group. NC, normal control; AM, ovalbumin immunization-induced asthmatic mice; BAM, BML-111-treated AM; VAM, vehicle of BML-111-treated AM; AAM, anti-IL-1β antibody-treated AM; RAM, rabbit IgG-treated AM; TLR, of toll-like receptor.

**Figure 5:**
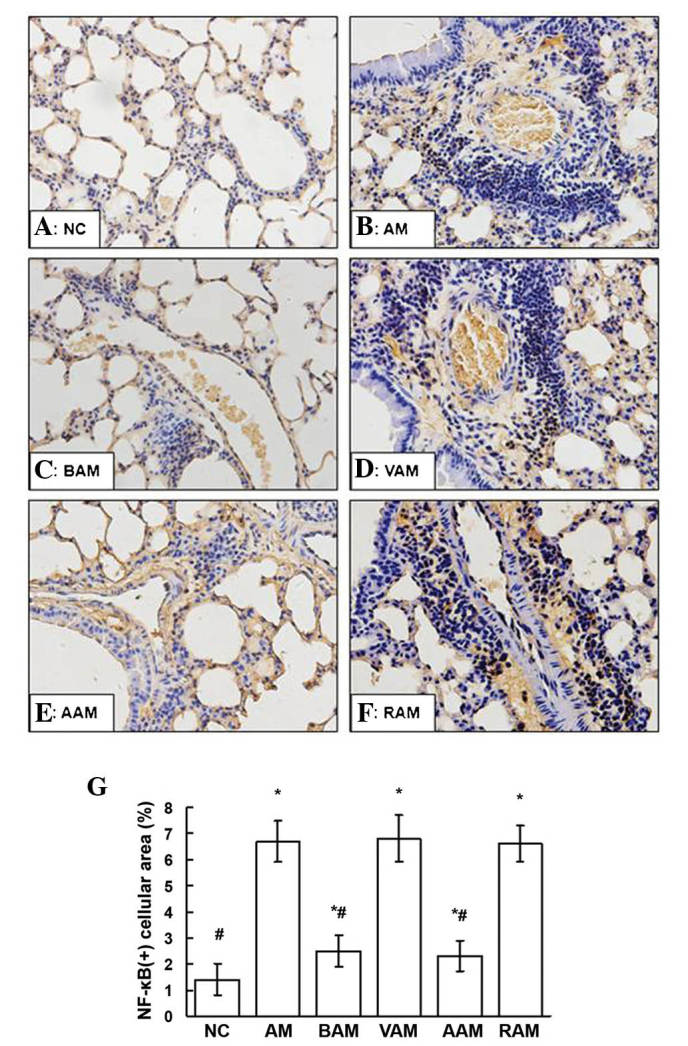
(A–F) Expression of NF-κB p65 in lung tissue assessed using immunohistochemistry in the mice in each group (deep brown cellular area indicates positive staining; magnification, x200). (G) The mean ratio of the NF-κB-positive cellular area in five fields of view per section of each mouse was assessed by JD-801 computer-aided image analyzer under high-power magnification (x200). Values are expressed as the mean ± standard deviation of five mice in each group. ^*^P<0.05, as compared with the NC group. ^#^P<0.05, as compared with the AM group. NC, normal controls; AM, ovalbumin immunization-induced asthmatic mice; BAM, BML-111-treated AM; VAM, vehicle of BML-111-treated AM; AAM, anti-IL-1β antibody-treated AM; RAM, rabbit immunoglob-ulin G-treated AM; NF-κB, nuclear factor-κB.

**Figure 6:**
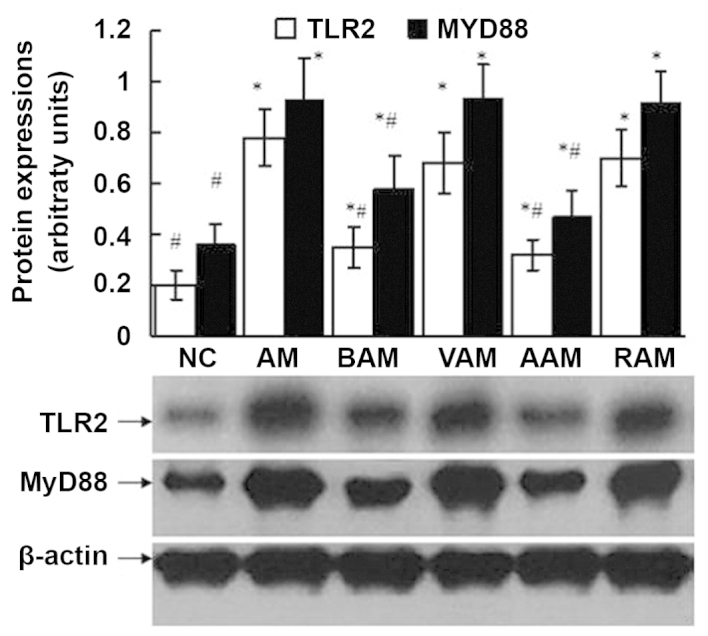
Expressions of TLR2 and MyD88 assessed using western blot analysis in lung tissue obtained from the mice in each group. The western blot shown is a representative of five independent experiments, and β-actin protein served as a loading control. Semiquantitative analysis was performed by using UVP-gel densitometry. Arbitrary unit = (A_TLR2_/A_β-actin_) × 100%, or (A_MyD88_/A_β-actin_) ×100%. Values are expressed as mean ± standard deviation of five mice in each group. ^*^P<0.05, as compared with the arbitrary units of the same protein in the NC group. ^#^P<0.05, as compared with the arbitrary units of the same protein in the AM group. NC, normal control; AM, ovalbumin immunization-induced asthmatic mice; BAM, BML-111-treated AM; VAM, vehicle of BML-111-treated AM; AAM, anti-IL-1β antibody-treated AM; RAM, rabbit immunoglobulin G-treated AM; MyD88, mycloid differentiation factor 88; TLR, toll-like receptor.

As shown in Figs. 4, 5 and 6, the expression of TLR2 and MyD88 assessed using western blot analysis and immunohistochemistry in the lungs signifi-cantly increased in the mice with AM compared with those in the normal controls. However, these elevated expression levels of TLR2 and MyD88 in the lungs induced by OVA were partially but significantly suppressed by BML-111 treatment (P<0.05). In a similar manner, treatment with anti-IL-1β antibody also inhibited the expression of TLR2 and MyD88 induced by OVA.

### Activity of NF-κB

**Figure 7:**
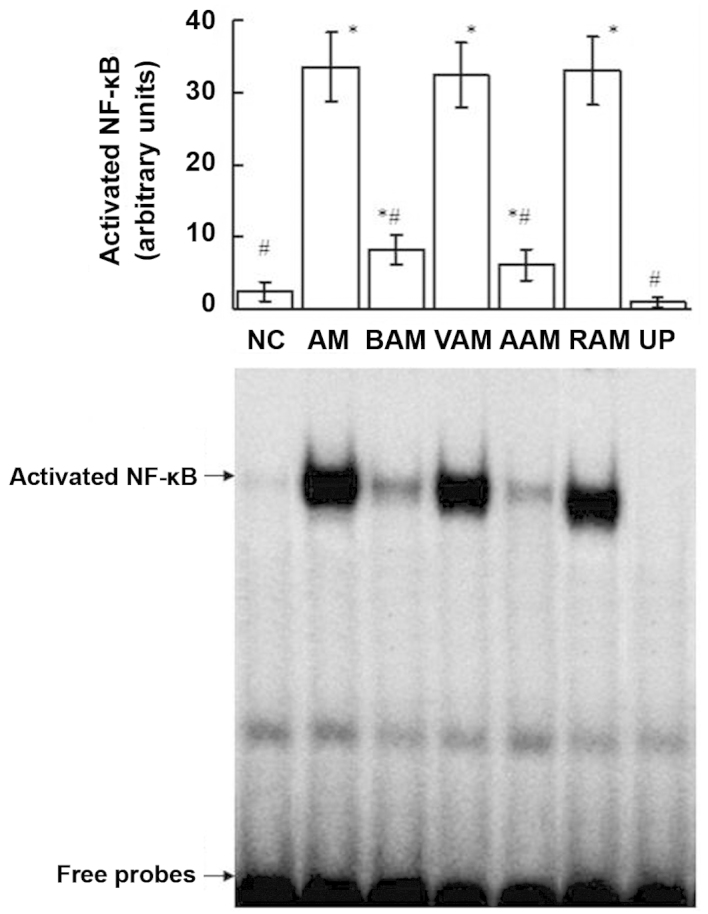
Activation of NF-κB assessed using EMSA in lung tissue obtained from the mice in each group. The EMSA gel is representative of five independent experiments. Semiquantitative analysis was performed by using UVP-gel densitometry. Arbitrary unit = (A_NC_ or A_AM_ or A_BAM_ or A_VAM_ or A_AAM_/A_unlabeled probes_) ×100%. Values are expressed as the mean ± standard deviation of five mice in each group. ^*^P<0.05, as compared with the arbitrary units of activated NF-κB in lungs obtained from NC mice. ^#^P<0.05, as compared with the arbitrary units of activated NF-κB in lungs obtained from AM mice. NC, normal control; AM, ovalbumin immunization-induced asthmatic mice; BAM: BML-111-treated AM; VAM, vehicle of BML-111-treated AM; AAM: anti-IL-1β antibody-treated AM; RAM, rabbit immunoglobulin G-treated AM; UP, unlabeled probes; NF-κB, nuclear factor-κB; EMSA, electrophoretic mobility shift assay.

As indicated in Fig. 7, OVA immunization upregulated the DNA-binding activities of NF-κB in the lungs. However, treatment with BML-111 significantly inhibited the activity of NF-κB induced by OVA. Additionally, treatment with anti-IL-1β antibody also suppressed the OVA-induced activity of NF-κB. The competition assay performed with unlabeled probes demonstrated the specificity of the DNA-binding activities of NF-κB detected using EMSA.

### Effects of BOC1, TLR2Ab, ST2825 and TPCA-1 on ILs released from leukocytes

**Figure 8:**
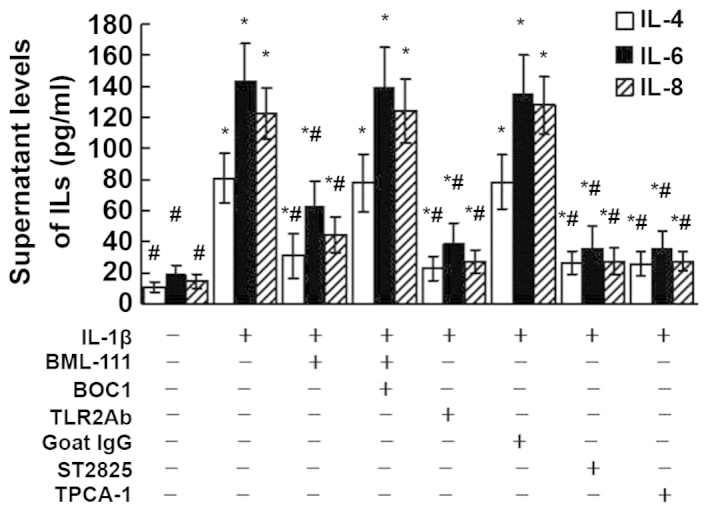
Supernatant levels of IL-4, IL-6 and IL-8 released from leukocytes exposed to IL-1β were assessed using ELISA. The cultured leukocytes were obtained from a mouse from the normal control group and stimulated with IL-1β (10 ng/ml) for 24 h with or without pre-treatment of BML-111 (1 mM) for 30 min, antagonist of G protein-coupled LXA4 receptor BOC1 (100 *μ*M) for 30 min, TLR2Ab (1 *μ*g/ml) for 1 h, goat IgG (1 *μ*g/ml) for 1 h, MyD88 dimerization inhibitor ST2825 (20 *μ*M) for 1 h and TPCA-1 (10 *μ*M) for 1 h. Values are expressed as the mean ± standard deviation of five independent experiments. ^*^P<0.05, as compared to the levels of same cytokine released from the cells without treatment. ^#^P<0.05, as compared to the levels of same cytokine released from the cells treated with IL-1β alone. IL, interleukin; IgG, immunoglobulin G; TLR, toll-like receptor; TPCA-1,10, thiophene-3-carboxamide 1; TLR2Ab, toll-like receptor 2-neutralizing antibody; BOC1, *N*-t-Boc-Phe-Leu-Phe-Leu-Phe.

As presented in Fig. 8, the levels of IL-4, IL-6 and IL-8 released from leukocytes increased following exposure of the cells to IL-1β. However, these enhanced IL levels were significantly diminished by BML-111 pre-treatment. These inhibitory effects of BML-111 were mediated by LXA4 receptor, as pre-treatment with BOC1 abolished the inhibitory effects of BML-111 on the IL hypersecretion induced by IL-1β. The enhanced levels of the ILs induced by IL-1β were also blocked by pre-treatment of the cells with TLR2Ab, ST2825 and TPCA-1.

### Effects of BOC1, TLR2Ab and ST2825 on the expression of TLR2 and MyD88 in leukocytes

**Figure 9:**
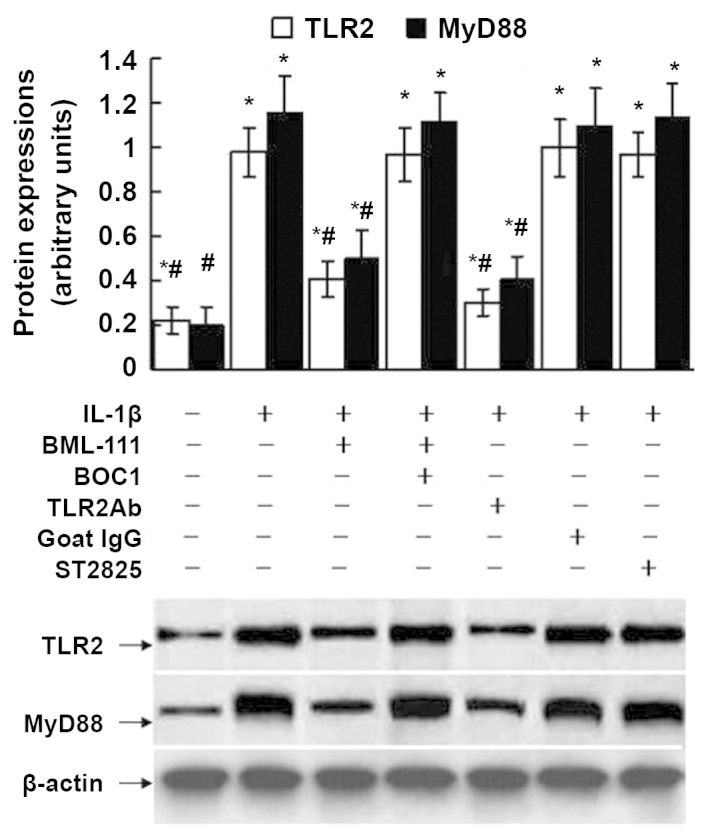
Expression of TLR2 and MyD88 assessed using western blot analysis of leukocytes exposed to IL-1β. The cultured leukocytes were obtained from a normal control mouse and stimulated with IL-1β (10 ng/ml) for 30 min with or without pre-treatment of BML-111 (1 mM) for 30 min, antagonist of G protein-coupled LXA4 receptor BOC1 (100 *μ*M) for 30 min, TLR2Ab (1 *μ*g/ml) for 1 h, goat IgG (1 *μ*g/ml) for 1 h and MyD88 dimerization inhibitor ST2825 (20 *μ*M) for 1 h. The western blot is representative of five independent experiments, and the lower panel shows β-actin protein, which served as a loading control. Semiquantitative analysis was performed by using UVP-gel densitometry. Arbitrary unit = (A_TLR2_/A_β-actin_) ×100%, or (A_MyD88_/A_β-actin_) ×100%. Values are expressed as the mean ± standard deviation of five independent experiments. ^*^P<0.05, as compared to the arbitrary units of the same protein in the cells without treatment. ^#^P<0.05, as compared to the arbitrary units of the same protein in the cells treated with IL-1β alone. IL, interleukin; IgG, immunoglobulin; TLR, toll-like receptor; TLR2Ab, toll-like receptor 2-neutralizing antibody; BOC1, *N*-t-Boc-Phe-Leu-Phe-Leu-Phe.

As shown in Fig. 9, treatment of the leukocytes with IL-1β augmented the expression of TLR2 and MyD88. However, these increments were alleviated by BML-111 pre-treatment. These inhibitory effects of BML-111 were mediated by the LXA4 receptor, as pretreatment with BOC1 abrogated the inhibitory effects of BML-111 on the expression of TLR2 and MyD88 induced by IL-1β. The IL-1β-triggered upregulation of TLR2 and MyD88 were repressed by TLR2Ab but not by ST2825. Since ST2825 is a dimerization inhibitor of MyD88 and inhibits the interaction of Flag-MyD88 and Myc-MyD88 proteins according to a previous co-immunoprecipitation assay (24), the total protein expression of MyD88 may be not affected by ST2825.

### Effects of BOC1, TLR2Ab, ST2825 and TPCA-1 on NF-κB activation in leukocytes

**Figure 10:**
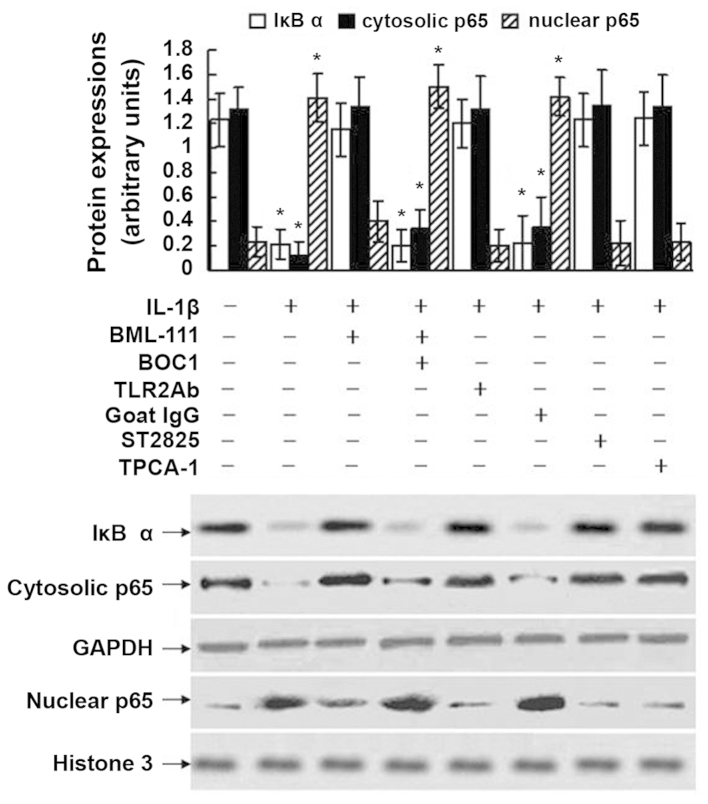
Activation of NF-κB assessed using western blot analysis in leukocytes exposed to IL-1β. The cultured leukocytes were obtained from a normal control mouse and stimulated with IL-1β (10 ng/ml) for 1 h with or without pre-treatment of BML-111 (1 mM) for 30 min, BOC1 (100 *μ*M) for 30 min, TLR2Ab (1 *μ*g/ml) for 1 h, goat IgG (1 *μ*g/ml) for 1 h, ST2825 (20 *μ*M) for 1 h or TPCA-1 (10 *μ*M) for 1 h. The western blot is representative of five independent experiments. GAPDH served as a loading control of cytosolic proteins. Histone 3 protein served as a loading control of nuclear proteins. Semiquantitative analysis was performed using UVP-gel densitometry. Arbitrary unit = (A_IκBα_/A_GAPDH_) ×100%, or (A_cytosolic p65_/A_GAPDH_) ×100%, or (A_nuclear p65_/A_histone 3_) ×100%. Values are expressed as the mean ± standard deviation of five independent experiments. ^*^P<0.05, as compared to the arbitrary units of the same protein in the cells without treatment. IL, interleukin; IgG, immunoglobulin G; TPCA-1,10, thiophene-3-carbox-amide 1; TLR2Ab, toll-like receptor 2-neutralizing antibody; BOC1, *N*-t-Boc-Phe-Leu-Phe-Leu-Phe; NF-κB, nuclear factor-κB; IκBα, inhibitor of kappa B alpha.

As indicated in Fig. 10, treatment of the leukocytes with IL-1β induced the degradation of IκBα and nuclear translocation of p65, as the levels of phosphorylated p65 subunit in the nuclear extract increased and the expression of p65 in the cytoplasmic extract decreased. However, BML-111 pre-treatment blocked the degradation of IκBα, as well as nuclear translocation of p65 induced by IL-1β. These inhibitory effects of BML-111 were mediated by the LXA4 receptor, as pretreatment with BOC1 abolished the inhibitory effects of BML-111 on the degradation of IκBα and nuclear translocation of p65 induced by IL-1β. The degradation of IκBα and nuclear translocation of p65 induced by IL-1β were also dramatically blocked by pretreatment of the cells with TLR2Ab, ST2825 and TPCA-1. LXA4 and its analog inhibited degradation, but not phosphorylation of IκBα in epithelial cells and keratinocytes (25,26), and in analogy with this, the present study detected degradation of IκBα but not phosphorylation of IκBα.

## Discussion

TLRs are important recognition receptors in the host innate defense against invading pathogens. TLR2 is particularly involved in signal transduction of cellular responses to lipoproteins/lipopeptides, Gram-positive bacteria and mycobacterial wall constituents (16). The IL-1 receptor (IL-1R) shares sequence homology with the cytosolic domain of the TLRs. The homologous cytoplasmic domain of IL-1R and TLRs is referred to as the Toll/IL-1R (TIR) homology domain (27,28). TLRs and IL-1R recruit the intracellular protein MyD88 via respective TIR domain interactions. These interactions result in the recruitment of IL-1R-associated kinase 1 (IRAK-1) to the receptor complex, where it interacts with tumor necrosis factor receptor-associated factor 6 (TRAF6), resulting in the downstream activation of NF-κB, which triggers the induction of a variety of effector genes, including genes of cytokines/mediators and ultimately results in upregulation of co-stimulatory molecules, secretion of cytokines, as well as enhanced uptake and presentation of antigen (16,27,28). The present study provided evidence that OVA-induced airway inflammation was mediated by upregulation of the TLR2/MyD88/NF-κB pathway. First, OVA immunization upregulated the protein expression of TLR2 and MyD88, and activated NF-κB in the lungs. Moreover, OVA-induced upregulation of the TLR2/MyD88/NF-κB pathway was further demonstrated by immunohistochemical staining in the lungs. Finally, consistent with the downregu-lation of TLR2/MyD88/NF-κB pathway proteins following treatment with BML-111- and anti-IL-1β antibodies, the OVA-induced airway inflammation was restrained, as shown by the reduced serum levels of IL-1β, IL-4 and IL-8, as well as BALF levels of IL-1β, IL-4, IL-8, OVA-IgE, and leukocyte counts; furthermore, peribronchial inflammation of lungs induced by OVA was ameliorated by BML-111- and anti-IL-1β antibodies. The findings of the present study are supported by those of other studies (17,29–32). For example, Lee *et al* (29) reported that TLR2 and TLR4 expression in lungs obtained from OVA-immunized mice was activated. Furthermore, in fatal asthma, elevated expression of TLR2, -3 and -4 in airways may have contributed to the acute inflammation causing asthma mortalities (29). Expression of TLR2 and TLR4 as well as that of the pro-inflammatory cytokines IL-8 and IL-1 increased in subjects with neutrophilic asthma as compared with that in other asthma subtypes and controls (31). In addition, activation of TLR2 induced a T helper type 2 immune response and promoted experimental asthma (17). Recently, it was demonstrated that organic dust extract-induced airway hyperresponsiveness, neutrophil influx and cytokine/chemokine production were nearly absent in MyD88 knockout mice, suggesting that acute organic dust-induced airway inflammatory response is highly dependent on MyD88 signaling, and is dictated by important contributions from upstream TLRs (32). In the present study, the ligands which activated the TLR2/MyD88/NF-κB pathway remain elusive; however, based on the *in vivo* and *in vitro* experiments, the most likely mediator was IL-1β.

As mentioned above, Toll/IL-1R is involved in the activation of several TLRs and the MyD88/NF-κB pathway. Therefore, the present study also explored the role of IL-1 in the pathogenesis of airway inflammation in OVA-immunized mice. The results of the present study demonstrated that IL-1β has a critical role in OVA-induced airway inflammation. First, the levels of IL-1β increased in the serum and BALF obtained from OVA-immunized mice. Treatment with anti-IL-1β antibody significantly suppressed the number of total leukocytes, eosinophils, neutrophils and lymphocytes in BALF stimulated by OVA; furthermore, it decreased the serum levels of IL-1β, IL-4 and IL-8, as well as the BALF levels of IL-1β, IL-4, IL-8 and OVA-IgE stimulated by OVA. Finally, treatment with anti-IL-1β antibody also blocked inflammatory cell infiltration and bronchial mucus hypersecretion induced by OVA. The results of the present study were supported by those of previous studies (33–37). For example, IL-1 is required for allergen-specific Th2 cell activation, synthesis of IgE and proinflammatory cytokines, and development of the airway hypersensitivity response (34–36). A recent study showed that inhalation of aerosolized anti-IL-1β antibody repressed the pathological responses in the pulmonary tissues of guinea pigs with asthma, and this inhibitory activity may be attributed to the decreased number of eosinophils and neutrophils and the reduced levels of inflammatory cytokines and IgE in the peripheral blood and BALF (37). The present study indicated that the inhibitory effects of anti-IL-1β antibody on OVA-induced airway inflammation may be mediated by the inhibitory role of anti-IL-1β antibody on the TLR2/MyD88/NF-κB pathway. First, treatment with anti-IL-1β antibody downregulated the expression of TLR2 and MyD88, and decreased the expression and activity of NF-κB in lungs induced by OVA. Secondly, IL-1β augmented the expression of TLR2 and MyD88 as well as the activity of NF-κB in leukocytes. Moreover, activation of NF-κB as well as enhanced levels of IL-4, IL-6 and IL-8 released from leukocytes exposed to IL-1β were blocked by TLR2Ab and ST2825. The results of the present study were supported by those of previous studies (24,38). For example, a previous study demonstrated that IL-1β stimulation significantly increased mRNA and protein expression of TLR2 and TLR4 in articular chondrocytes (38). Moreover, the MyD88 inhibitor ST2825 blocked IL-1β-induced synthesis of IL-6 *in vivo* as well as activation of NF-κB in treated mice, indicating that TLRs/MyD88 mediated IL-1β-induced, NF-κB-dependent production of IL-6 (24).

Signaling pathways involved in LXA4 activity in asthma remain elusive, although it was demonstrated that an LXA4 analog blocked airway hyper-responsiveness and pulmonary inflammation in a murine model of asthma (11). The present study showed that LXA4 receptor activation by BML-111 inhibited the production of IL-1β, IL-4 and IL-8 in serum and BALF, restored the production of IFN-γ in serum obtained from OVA-immunized mice, and suppressed airway inflammation. These effects of BML-111 may be attributed to the BML-111-induced downregulation of the TLR2/MyD88/NF-κB pathway. First, BML-111 treatment downregulated the expression of TLR2 and MyD88, as well as the expression and activity of NF-κB in the lungs induced by OVA. Consistent with BML-111-induced downregulation of the TLR2/MyD88/NF-κB pathway, the OVA-induced airway inflammation was restrained, as shown by the reduced serum levels of IL-1β, IL-4 and IL-8, as well as BALF levels of IL-1β, IL-4, IL-8, OVA-IgE. Furthermore, leukocyte counts were reduced and peribronchial inflammation was ameliorated in lungs induced by OVA. Finally, the elevated levels of IL-4, IL-6 and IL-8, expression of TLR2 and MyD88, and activation of NF-κB in leukocytes were all diminished by BML-111. In the present study, BML-111-induced downregulation of the TLR2/MyD88/NF-κB pathway in OVA-immunized mice may be attributed to the following reasons: First, BML-111 itself downregulated the TLR2/MyD88/NF-κB pathway; second, BML-111 reduced the levels of IL-1β in the serum and BLAF, and therefore inhibited the IL-1β-induced upregulation of the TLR2/MyD88/NF-κB pathway; and third, BML-111 decreased the pulmonary infiltration of leukocytes and this decrement in the number of total cells in the lungs may have contributed to the reduced protein expression of the TLR2/MyD88/NF-κB pathway in the lung tissue.

In conclusion, the present study identified that first, OVA-induced airway inflammation is mediated by upregulation of the TLR2/MyD88/NF-κB pathway; second, IL-1β has a pivotal role in the airway inflammation and upregulation of the TLR2/MyD88/NF-κB pathway induced by OVA; and third, LXA4 receptor activation by BML-111 restrains the OVA-induced airway inflammation via downregulation of the TLR2/MyD88/NF-κB pathway. Coupled with a previous demonstration of the efficacy of LXA4 and LXA4 analog in the treatment of asthmatic mice and humans (11,39), the present study provided a theoretical basis and supported a therapeutic value for clinical use of LXA4 analogs in the treatment of asthma. The efficacy of anti-IL-1β antibody was previously proven in the treatment of asthmatic guinea pigs (37), and the present study also supports a therapeutic value for anti-IL-1β antibody in the treatment of refractory asthma.
